# Analysis of the Genetic Mechanism of Yield-Related Traits of Maize in Cold Regions

**DOI:** 10.3390/genes16080941

**Published:** 2025-08-08

**Authors:** Chao Gao, Zimeng Li, Guogang Zheng, Hong Di, Lin Zhang, Zhenhua Wang, Ling Dong

**Affiliations:** College of Agriculture, Northeast Agricultural University, Harbin 150030, China; gc203203@126.com (C.G.); lzm072811@163.com (Z.L.); 15806156347@163.com (G.Z.); dihongdh@163.com (H.D.); neauzla@163.com (L.Z.); zhenhuawang_2006@163.com (Z.W.)

**Keywords:** agronomy, maize, combining ability, diallel cross, genetic

## Abstract

Background: Maize is an important food crop in cold regions, especially in Northeast China. However, its short growth period and low-temperature stress pose challenges to the breeding of high-yield hybrids. With climate warming, the maize planting area continues to expand to high latitudes. Research on cold-region maize is of great significance to ensure food security and sustainable agricultural development. However, most of the current maize research is concentrated in temperate and tropical regions, and there are few studies on cold-region maize. Methods: Based on this, this study selected some representative cold-region maize materials and materials whose adaptability has not yet been verified, and used a semi-diallel hybrid design for hybridization to determine the general combining ability (GCA) and specific combining ability (SCA) to screen out excellent breeding materials suitable for cold regions. Field experiments were carried out under four different cold environments, and 55 hybrid progenies and their parents were evaluated. The double allele hybridization analysis based on the Griffing method 2 (model 1) showed that the specific combining ability (SCA) and general combining ability (GCA) effects of each trait were significant. Results: The GCA mean square of all traits except yield and number of grains per row was greater than the SCA mean square, indicating that additive gene effects were dominant and genetic improvement through selective breeding was feasible. Hayman plot analysis under four environments showed that yield, ear length, number of grains per row, water content, and plant height were mainly controlled by superdominant genes, while stem thickness, number of nodes, and ear position were controlled by some dominant genes. Conclusions: Parent P1 contained more recessive genes in yield traits, but more dominant genes in number of grains per row, number of nodes, and ear position; P3 contained more dominant genes in yield and water content, but more recessive genes in number of nodes and ear position; P7 contained more recessive genes in most traits; and P9 contained more dominant genes in most traits. P9 and P6 represent excellent parental germplasm, among which the hybrid combinations P1 × P9, P2 × P5, P3 × P10, P4 × P6, P5 × P8, P6 × P9, P7 × P10, and P8 × P10 all show hybrid vigor exceeding that of their parents and have high breeding value.

## 1. Introduction

Maize (*Zea mays* L.) ranks among the world’s most important cereal crops, serving both human consumption and livestock feed demands [1]. Given the tremendous demand for maize in poultry and livestock feed as well as human consumption, high-yielding, high-quality hybrid maize breeding programs have long been underway [2]. Although yields have climbed steadily since the late 20th century, many of the world’s major production regions have recently seen a deceleration or plateau in yield gains—largely due to the homogenization of genetic backgrounds in commercial cultivars and the erosion of germplasm diversity [3,4]. The key to achieving a breakthrough in yield lies in employing genetic approaches to dissect the modes of gene action underlying yield and its component traits (such as plant height, kernel moisture content, and ear height), and, through the coordinated improvement of multiple traits, formulating an integrated, scientifically based breeding strategy to fully realize its potential. At the same time, in the context of global climate change, cold-region maize is gradually becoming an important field in agricultural research and practice. As the climate warms, maize-growing areas continue to expand to high-latitude areas. Therefore, conducting cold-region maize research is of great significance to ensuring food security and sustainable agricultural development [5].

As global climate change intensifies, agricultural production in cold and high-altitude areas faces many challenges. Qian [6] studied the yield changes of spring wheat, canola, and maize in Canada under different global warming scenarios and found that crop yields generally decreased with rising temperatures, especially in high-latitude areas. In the highlands of Peru, maize varieties such as “Chullpi” grow well in cold climates at altitudes between 2400 and 3400 m, demonstrating the adaptability of maize in high-altitude areas [7].

Maize in cold regions faces low-temperature stress, which may lead to delayed germination and slow growth, affecting yield. In contrast, maize varieties in warm regions focus more on characteristics such as drought resistance and disease resistance. Therefore, studying the cultivation technology and variety improvement of cold-region maize will not only help improve food security in cold regions but also provide an important reference for agricultural adaptability in the context of global climate change.

The diallel mating-analysis framework is an effective tool for elucidating the genetic control patterns of key quantitative traits among a set of parental lines, and it aids breeders in selecting superior parents and devising subsequent breeding schemes [8,9]. Diallel mating designs include the full diallel and the half diallel. The half-diallel design conducts only all possible crosses among parents (excluding reciprocals), substantially reducing the experimental scale while still ensuring accurate estimation of genetic effects [10]. Matzinger et al. employed diallel analysis across multiple locations and years to elucidate the effects of genotype × environment interactions on combining-ability estimates, thereby laying the methodological foundation for multi-environment combining-ability research [11].

Hayman (1954) [12] and Griffing (1956) [10] each provided a comprehensive framework for the separate estimation of gene action and combining ability. Griffing’s (1956) classic method for partitioning general combining ability (GCA) and specific combining ability (SCA) remains widely applied in genetic research and breeding practice in maize and other crops [10]. In Griffing’s analysis, the total variance is partitioned into GCA and SCA components, which respectively reflect the contributions of additive and non-additive gene effects to trait expression. Specifically, GCA measures a parent’s average genetic contribution—capturing primarily additive variation—while SCA quantifies the deviation of a specific cross from parental means, indicating the strength of dominance and overdominance interaction effects [10]. Research has shown that parental combinations derived from markedly divergent genetic backgrounds, when endowed with both high general combining ability (GCA) and specific combining ability (SCA), often exhibit pronounced heterosis and yield-enhancement potential in the F_1_ generation [13]. Meanwhile, Hayman (1954), by partitioning additive variance (D) and dominance variances (H_1_, H_2_) and employing Vr–Wr regression-graph analysis, profoundly elucidated the patterns of additive, dominance and overdominance gene action in the inheritance of quantitative traits, thereby providing a robust basis for investigations into the genetic mechanisms underlying quantitative traits [12]. By integrating the Griffing and Hayman analytical frameworks, breeders gain a comprehensive theoretical foundation for identifying parental lines and hybrid combinations that simultaneously exhibit high GCA and high SCA, as well as for formulating efficient breeding strategies. This combined approach is therefore critical for optimizing parent selection and constructing highly effective breeding programs [14].

At present, most studies on the genetic mechanism of maize are concentrated in temperate and tropical regions, while there are few research data on cold-region maize. Based on this background, this study selected 10 cold-region maize materials and materials whose adaptability has not yet been verified, and used a 10 × 10 semi-diallel hybrid design for hybridization. Field trials were conducted in Suihua and Huanan during the growing seasons of 2017 and 2018 to measure key agronomic traits. Multivariate analysis of variance was used to estimate general combining ability (GCA), specific combining ability (SCA), and genetic variation components, combined with Vr–Wr regression and genetic parameter analysis.

This study proposes the following hypotheses: (1): It is assumed that the main agronomic traits of cold-region maize have significant genetic differences under different parental combinations, and through reasonable hybrid combinations, hybrid offspring with excellent performance can be obtained. (2): It is assumed that the main agronomic traits of cold-region maize can be effectively analyzed by the Griffing and Hayman methods to analyze the genetic control mode of these gene effects, thereby providing a theoretical basis for the variety improvement of cold-region maize. (3): It is assumed that parental combinations from different genetic backgrounds can show strong heterosis in cold-region environments, and can obtain hybrids with excellent performance by selecting parents with high total combining ability (GCA) and specific combining ability (SCA). The research objectives include (1) evaluating the general combining ability (GCA) and specific combining ability (SCA) of major agronomic traits in cold-region environments; (2) analyzing the genetic control mode of traits using the Griffing method and the Hayman method; and (3) screening out excellent parents and excellent combinations suitable for cold-region breeding applications.

## 2. Materials and Methods

### 2.1. Experimental Location and Climate

The field trials were conducted in four different locations/year environments in 2022 and 2023, with two sites in Northeast China: Suihua and Huanan. Specifically, the trials in Suihua are ENV1 (2022) and ENV2 (2023), and the trials in Huanan are ENV3 (2022) and ENV4 (2023). Both regions belong to cold-region maize growing areas, with cold winters and short summers, and typical climatic characteristics, providing environmental conditions suitable for cold-region maize research. In terms of field management, the trials were conducted in accordance with standard operations of local agricultural production to ensure the scientificity and consistency of the experiments.

### 2.2. Breeding Materials

Ten parental lines, including cold-region maize varieties and cold-tolerant potential materials (Table 1), were selected in this study to provide a diverse genetic basis for breeding programs. These parental lines include P1 (K10), P2 (KF298), P3 (Dong 46), P4 (Jing 7), and P5 (He 344), all of which are cold-region maize or cold-tolerant (potential) varieties. In addition, P6 (DE1), P7 (DE3, female parent), P8 (DE3, male parent), P9 (FA5-1), and P10 (FA5-5) represent different cold-region maize varieties. The selection of these ten parental lines enabled us to establish a 10 × 10 semi-diallel hybridization scheme to generate all possible hybrid combinations except reciprocal crosses. This diverse set of materials aims to deeply explore the genetic mechanisms of yield-related traits in cold-region maize and lay a solid foundation for future breeding work.

### 2.3. Data Collection

At physiological maturity, nine traits were measured for all entries in each of the four environments: ear length, stalk diameter, kernel rows per ear, number of stem nodes, number of husk leaves, grain yield, moisture content, ear height, and plant height. Both the parents and their F_1_ hybrids in the half-diallel design were laid out with three replicates per environment, and the check varieties were similarly replicated three times. Within each replicate plot, five vigorous plants were selected from interior rows (excluding border rows) for trait measurements, and the mean of these five plants was used as the phenotype value for that replicate.

### 2.4. Statistical Analysis

All data analysis and drawing were completed using R 4.3.2.

#### 2.4.1. Griffing’s Combining Ability Analysis

Griffing’s [10] model 2, method 1 was used to analyze general combining ability (GCA) and specific combining ability (SCA) and to estimate their respective variance components. This analytical framework is tailored for half-diallel mating designs that include parental self-crosses. The mathematical model is as follows:

(1) $$x_{ij}=u+g_{i}+g_{j}+S_{ij}+\gamma_{\dot{z}j}+\frac{1}{b}\Sigma bk+\frac{1}{b}{\left(bv\right)}_{ijk}+\frac{1}{bc}\sum_{kl}\Sigma e_{ijkl}$$

The ratio of combining abilities was calculated using Baker’s method as follows:

(2) $$\mathrm{Baker}\;\mathrm{ratio}=\frac{2{MS}_{GCA}}{2{MS}_{GCA}+{MS}_{SCA}}$$

Here, ${MS}_{GCA}$ and ${MS}_{SCA}$ denote the mean squares for general combining ability and specific combining ability, respectively.

#### 2.4.2. Hayman’s Genetic Analysis Method

Analysis of variance for F_1_ progeny in a diallel mating design [8]:

(3) $$Y_{ijkl}=m+T_{ij}+b_{k}+(bT{)}_{ijk}+e_{ijkl}$$

$Y_{ijkl}$ is the observed value of the ith and jth parents in the kth replicate on the lth individual; m is the overall mean; $T_{ij}$ is the genotypic effect of the i × j combination; $b_{k}$ is the effect of the kth replicate; $(bT{)}_{ijk}$ is the interaction effect between the kth replicate and the genotype of the i × j combination; and $e_{ijkl}$ is the error effect.

The genetic variance components were calculated using the following formula:

(4) $$b\left(W_{r},V_{r}\right)=\frac{Cov\left(W_{r},V_{r}\right)}{Var\left(V_{r}\right)}$$

Here, $W_{r}$ denotes the covariance between parents and offspring; $V_{r}$ denotes the variance of each array;$\;Cov\left(W_{r},V_{r}\right)$ denotes the covariance of $\left(W_{r},V_{r}\right)$; and $Var\left(V_{r}\right)$ denotes the variance of $\left(V_{r}\right)$.

The dominance variance D was calculated as follows:

(5) $$D=V_{0L0}-E$$

Here, D denotes the dominance genetic variance component, $V_{0L0}$ represents the variance among parents, and E represents the environmental variance.

The dominance variance $H_{1}$ was calculated as follows:

(6) $$H_{1}=V_{0L0}-4W_{0L01}+4V_{1L1}-\frac{\left(3n-2\right)E}{n}$$

In the equation, $V_{0L0}$ is the parental variance; $W_{0L01}$ is the covariance between parents and arrays; $V_{1L1}$ is the variance of array means; n is the number of parents; and E is the environmental variance.

Proportion of positive-effect versus negative-effect genes in the parental lines:

(7) $$H_{2}=4V_{1L1}-4V_{0L1}-2E$$

$V_{1L1}$ denotes the variance of the array means, $V_{0L1}$ denotes the variance of the parental means, and E denotes the environmental variance.

Average covariance between additive and dominance effects:

(8) $$F=2V_{0L0}-4W_{0L0}-\frac{2\left(n-2\right)E}{n}$$

In the equation, $V_{0L0}$ denotes the parental variance; $W_{0L0}$ denotes the covariance between parents and arrays; n is the number of parents; and E is the environmental variance.

Dominance effect:

(9) $$h^{2}=\frac{4{\left(M_{L1}-M_{L0}\right)}^{2}-4(n-1)E}{n^{2}}$$

In the equation, ${\left(M_{L1}-M_{L0}\right)}^{2}$ represents the difference between the parental mean and the mean of its n offspring; n is the number of parents; and E is the environmental variance.

The regression coefficient was calculated as follows:

(10) $$b={\left[\frac{\left\{Var\left(W_{r}\right)-bCov\left(W_{r},V_{r}\right)\right\}}{Var\left(V_{r}\right)\left(n-2\right)}\right]}^{1/2}$$

In the equation, $W_{r}$ denotes the covariance between parents and offspring; $V_{r}$ denotes the variance of each array; $Cov\left(W_{r},V_{r}\right)$ denotes the covariance of $\left(W_{r},V_{r}\right)$; and $Var\left(V_{r}\right)$ denotes the variance of $\left(V_{r}\right)$.

The significance tests for whether the regression coefficient b differs from zero and unity are as follows:

(11) $$Ho:b=0=\frac{\left(b-0\right)}{SE\left(b\right)}$$

(12) $$Ho:b=1=\frac{\left(1-b\right)}{SE\left(b\right)}$$

(13) $$t^{2}=\frac{\left(n-2\right)}{4}\times \frac{{\left(VarVr-VarWr\right)}^{2}}{VarVr\times VarWr-{Cov}^{2}\left(Vr,Wr\right)}$$

The estimated regression coefficients were tested against the critical t-values with n − 2 degrees of freedom. Vr–Wr analysis revealed that the regression coefficient b deviated significantly from unity, implying the presence of non-allelic interactions; if b does not differ significantly from 1, it indicates that such interactions do not contribute to trait inheritance. A $t^{2}$ test for the uniformity of Vr–Wr values was also conducted to validate the assumptions of the diallel analysis.

Correlation coefficient between WR and VR:

(14) $$\frac{Cov\left(W_{r}+V_{r}\right),Y_{r}}{\sqrt{Var\left(W_{r}+Y_{r}\right)\cdot Var\left(Y_{r}\right)}},$$

$\left(W_{r}+Y_{r}\right)$ denotes the ranking of parental dominance degree; $Y_{r}$ denotes the observed value of the parent; $Cov\left(W_{r}+V_{r}\right)$ denotes the covariance between $\left(W_{r}+Y_{r}\right)$; $Var\left(W_{r}+Y_{r}\right)$ denotes the variance of $\left(W_{r}+Y_{r}\right)$; and $Var\left(Y_{r}\right)$ denotes the variance of $Y_{r}$.

Narrow-sense heritability:

(15) $$h_{N}^{2}=\frac{\frac{1}{2}D+\frac{1}{2}H_{1}-\frac{1}{2}H_{2}-\frac{1}{2}F}{\frac{1}{2}D+\frac{1}{2}H_{1}-\frac{1}{4}H_{2}-\frac{1}{2}F+E}$$

In the equation, D denotes the additive genetic variance, $H_{1}$ denotes the dominance variance, $H_{2}$ denotes the proportion of positive-effect or negative-effect genes in the parental lines, F denotes the average covariance between additive and dominance effects, and E denotes the environmental variance.

## 3. Results

### 3.1. Variation of All Genotypes in the Combined Environments

As shown in Table 2, except for ear length and stalk diameter, all measured traits differed highly significantly (*p* ≤ 0.01) across environments and genotypes (including parents and progeny). In the combined data for the ten parents and their hybrids over four environments, the genotype × environment (G × E) interactions for husk leaf number, ear height, and plant height were also highly significant (*p* ≤ 0.01). The coefficients of variation (CV%) ranged from 2.82% to 63.55%, indicating a wide spectrum of trait variability.

### 3.2. Average Performance of Genotypes Across Four Environments

Table 3 shows the average performance of all parental genotypes in the cold environment. Plant height ranged from 142.2 to 227.8 cm, with parent P2 having the lowest average and P8 the highest. Grain yield varied widely, peaking at 7.26 t/hm^2^ for P1 and dropping to 4.52 t/hm^2^ for P10. Grain moisture content fluctuated between 21.27% (P5) and 25.56% (P9), with P5’s low moisture suggesting better suitability for mechanical harvesting or dehydration. Ear height showed clear architectural differences among genotypes, from 50.98 cm in P2 up to 82.70 cm in P9. Ear length varied little, from 13.84 cm (P4) to 16.82 cm (P7), and the number of husk leaves ranged from 8.57 (P7) to 9.90 (P6). Stalk diameter was thinnest in P3 (19.94 mm) and thickest in P8 (22.66 mm), implying stronger lodging resistance in P8. The number of stem nodes spanned from 10.40 (P1) to 11.92 (P3), and higher counts in genotypes like P3 and P9 may reflect longer growth periods or greater biomass accumulation. Kernel row number was highest in P9 (29.49) and also strong in P5 and P7 (around 29 rows), which could enhance single-ear yield.

### 3.3. Combining Ability Analysis of Traits in Maize Hybrids

#### 3.3.1. Analysis of Combining Ability Effects Using Griffing’s Method

Combining ability variance analysis was performed using Griffing’s method [10] for all traits analyzed in the combined four-environment dataset (see Table 4). General combining ability (GCA) is generally regarded as representing additive genetic effects and the additive component of dominance variance; by contrast, specific combining ability (SCA) reflects non-additive genetic effects and the remaining dominance variance [11]. The highly significant GCA and SCA effects across all traits indicate that parental performance is governed by both additive and non-additive gene action. The GCA and environment interactions of all traits except stem length, plant height, and stem diameter reached significant or extremely significant levels, indicating that the GCA of inbred lines was greatly affected by environmental conditions. On the other hand, the SCA and environment interactions of all traits except the number of bracts did not reach significant levels, indicating that the response of SCA to the environment was relatively stable and not easily affected by environmental changes. Therefore, the additive effect has a large change in adaptability to different environments, while the non-additive effect is relatively stable under different environments. For all traits except grain yield and kernel rows per ear, GCA mean squares exceeded those of SCA, demonstrating the predominance of additive effects across the four environments. Baker’s ratios ranged from 0.57 to 0.94, further underscoring the pivotal role of additive effects in trait inheritance.

#### 3.3.2. Analysis of General and Specific Combining Ability

Maize plant height is influenced by the combined effects of stem node number and internode length; thus, the number of stem nodes is significantly positively correlated with plant height [15]. In the combined data (Table 5), parents P2, P6, and P4 showed negative GCA for stem node number and plant height, indicating they tend to pass on shorter plant stature in hybrids; these lines could serve as parents for breeding short-stature maize. Parent P9 had significant positive GCA effects (*p* < 0.01) for yield (0.86), grain moisture content (0.73), plant height (4.65), and ear height (4.66). Besides P9, P8 also showed a positive GCA for yield, though not significant. Parents P3, P5, and P6 had significant positive GCA for ear length, while P4 and P10 had significant negative effects. Parent P6 showed a significant positive GCA for husk leaf number, tending to transmit more husk leaves to its progeny. Parent P10 exhibited highly significant negative GCA effects for yield (–0.57), ear length (–0.46), husk leaf number (–0.36), and kernel rows (–1.16).

Table 6 summarizes the SCA effects of 45 hybrid combinations across environments. For plant height, 43 combinations showed significantly positive SCA effects, led by P2 × P10 (31.26), followed by P5 × P10 (25.11), P1 × P2 (22.82), and P1 × P10 (21.83), all significant at *p* < 0.01, indicating strong heterotic effects on height. Stalk diameter SCA effects ranged from –0.89 to 1.98; among 30 positive-effect crosses, P2 × P9 (1.98) was highest, then P3 × P9 (1.48) and P1 × P9 (1.47), all *p* < 0.01. For the stem node number, 25 crosses had significant SCA effects: P1 × P10 (2.18) was highest, followed by P8 × P9 (1.93) and P2 × P5 (0.45); conversely, P5 × P6 (–0.41) and P9 × P10 (–0.34) showed highly significant negative effects (*p* < 0.01), potentially useful for compact plant types. Husk leaf number SCA ranged from –0.55 to 1.56, with P1 × P8 (1.56) highest; P6 × P9 (1.30) and P4 × P6 (0.84) were also significant. Kernel row number was highest in P3 × P8 (3.34), followed by P1 × P5 (3.11) and P1 × P7 (2.86), all highly significant, demonstrating advantages for single-ear grain number. Ear length SCA effects spanned –0.42 to 1.35, led by P1 × P5 (1.35), then P4 × P6 (1.29), P3 × P7 (1.22), and P2 × P5 (0.97). Grain moisture SCA ranged from –1.94 to 2.52; 18 crosses were negative, with P6 × P10 (–1.15), P1 × P2 (–0.89), and P7 × P8 (–0.84) most significant. Yield SCA effects varied from –1.92 to 2.61; 35 crosses were positive, with P3 × P9 (2.39), P6 × P10 (2.30), and P1 × P7 (2.21) highest and significant (*p* < 0.01), marking them as potential high-yield hybrids, while P5 × P6 (–1.83) and P3 × P6 (–1.37) with significant negative effects may be suited to dwarf or yield-controlled breeding.

### 3.4. Hayman Variance Analysis and Estimation of Genetic Components

This study employed Hayman’s method to dissect the genetic variance components and their proportions for each evaluated maize trait (Table 7). For all traits examined, the dominance variance component (H_1_) exceeded the additive variance (D). Moreover, seven traits exhibited significantly negative dominance variances (H_1_ and H_2_), whose absolute magnitudes surpassed the additive variance (D), indicating that these traits are chiefly governed by non-additive genetic effects and involve deleterious dominance or overdominance interactions. For six of the traits, the primary dominance variance (H_1_) exceeded the secondary dominance variance (H_2_), indicating an asymmetric distribution of allele frequencies among the parental lines and suggesting that dominance inheritance is prevalent for these traits [16]. The findings of T. Sandeep Kumar on ear length and kernels per row are consistent with those of the present study [17]. Of the nine traits evaluated, seven—namely grain yield, husk leaf number, ear length, stem diameter, kernels per row, node number, and grain moisture content—exhibited significantly negative F-values, indicating that dominant–recessive gene pairs in the parental lines have a substantial genetic basis and impact on these traits. In contrast, the F-values for ear position and plant height were positive, suggesting that these traits are primarily governed by dominant alleles.

The difference between H_1_ and H_2_ indicates that the distribution of dominant and recessive genes is uneven. Since the H_2_/4H_1_ ratio of seven out of the nine traits studied is lower than the theoretical value of 0.25, it indicates that the positive and negative allele frequencies of the parents in these traits are asymmetric, and there may be an uneven distribution of dominant or recessive alleles. The H_2_/4H_1_ ratio of ear position is 0.25, and the H_2_/4H_1_ ratio of plant height is close to 0.25, indicating that the distribution of dominant and recessive genes in the parents for these two traits is relatively symmetrical, which is consistent with the gene frequency distribution expected by theory. In addition, the importance of phenotypic traits being affected by non-additive gene effects has been further verified, and the broad heritability (h^2^) of the traits of six traits is at a low or medium level. By estimating the (H_1_/D)^1/2 ratio, it was found that the ratio was greater than 1 in all nine traits studied, indicating that the average dominance of these traits was high and that overdominant gene effects were common in inheritance. The above results are consistent with the distribution trend of the WR-VR graph, further verifying the dominant role of non-additive genetic components in these target traits.

### 3.5. Vr–Wr Regression Analysis

**Figure 1:**
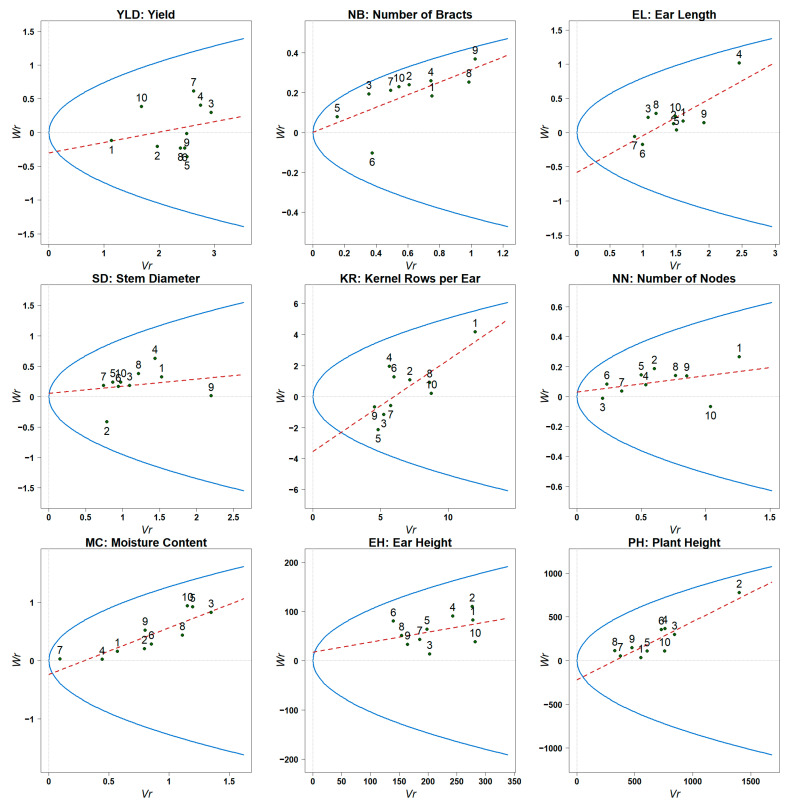
Regression analysis of WR and VR of each trait of 10 parents; 1–10 represent P1–P10.

Table 8 and Figure 1 shows the scatter plot of parent–offspring covariance (Wr) and variance (Vr) for each trait and its regression results; for grain moisture, based on all 10 parents, the regression coefficient b is significantly greater than 0 (reject H_0_: b ≤ 0), and is not significantly different from 1 (failed to reject H_0_: b = 1), indicating that the trait follows a simple additive–dominant genetic model and there is no overdominance or non-allelic interaction; for bract number, ear length, number of kernels per row and plant height, in the F_1_ generation, b deviates significantly from both 0 and 1, indicating that it is neither a pure additive model nor a pure dominant model, and there is allelic interaction (overdominance); for the remaining traits, b is significantly different from 1, but not significantly different from 0, indicating that they may be controlled by overdominant genes or non-allelic interactions.

#### 3.5.1. Average Dominance

The deviation of the intercept of the Vr-Wr regression line reveals the dominance patterns of different quantitative traits; the intercepts of yield, ear length, number of grains per row, water content and plant height are all below the origin, indicating that these traits are dominated by overdominant genes; the intercepts of stem thickness, number of stem nodes and ear position are above the origin, indicating that they are incompletely dominant; the intercept of bract number is zero, suggesting that this trait follows an additive or intermediate dominance pattern; and the intercepts of growth traits such as ear height and stem thickness are close to but slightly above, which also supports a mixed genetic effect dominated by partial dominance or additiveness.

#### 3.5.2. Parental Inheritance of Dominant and Recessive Genes

By analyzing the Vr–Wr regression graph, the distribution characteristics of dominant and recessive alleles of each parent can be comprehensively evaluated [12,18]. Generally speaking, parents close to the origin in the Vr–Wr graph carry dominant alleles with a higher frequency, while parents farther from the origin have dominant recessive genes. In this study, P1 was closest to the origin in yield traits, but was far away from the origin in the number of grains per row, number of nodes, and ear position traits. P3 was far away from the origin in yield and water content traits, but close to the origin in the number of nodes and ear position traits. Compared with other parents, P7 was closer to the origin in most traits, indicating that it contained more dominant alleles in the relevant traits. P9 showed a trend of being far away from the origin in most traits, indicating that it had a higher content of recessive genes. In addition, the correlation coefficient r between Wr + Vr and the trait mean Yr can be used to determine the direction of the dominant gene effect (Table 7); a positive r value means that the dominant allele generally reduces the trait expression (negative effect), and a negative r value indicates that the dominant gene enhances the trait value (positive effect) [12,19]. The correlation coefficients of Wr + Vr and Yr for all traits in this study were negative, indicating that the dominant alleles of the parents generally have a positive gain effect on each trait.

### 3.6. Calculation of Excess Advantage

**Figure 2:**
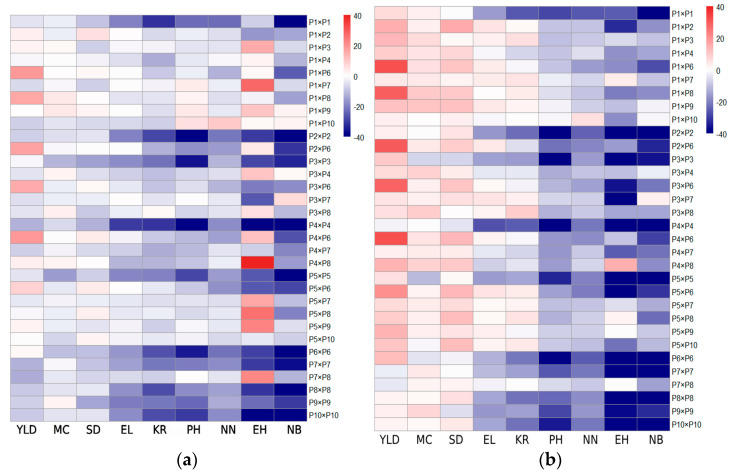
Advantages of exceeding the standard. (**a**) Zhedan 37 comparison; (**b**) Demeiya 3 comparison EL: ear length; SD: stem diameter; KR: kernel rows per ear; NN: number of nodes; NB: number of bracts; YLD: yield; MC: moisture content; EH: ear height; PH: plant height.

By comparing the varieties Zhedan 37 and Demeiya 3, the standard heterosis of different parental combinations was evaluated on nine major traits (Figure 2), providing an intuitive basis for new variety selection and genetic improvement strategies. Taking Zhedan 37 as a reference, hybrid combinations such as P1 × P6, P1 × P8, and P3 × P7 showed significant positive heterosis in plot yield (PY) and grain number per row (GPR) (see red blocks in the heat map); similarly, in the background of Demeiya 3, combinations such as P1 × P7, P2 × P6, and P4 × P4 also showed considerable yield gains. In contrast, inbred lines P2 × P2 and P3 × P3 showed negative heterosis (dark blue blocks) in traits such as plant height (PH), panicle length (SL), and bract wrapping (BL) in both controls, indicating the presence of typical inbreeding depression. The comprehensive heterogeneous advantage heat map analysis of nine traits not only identified excellent outcrossing combinations with stable gains such as P1 × P6, P3 × P7, and P2 × P6, but also clarified the characteristic manifestations of self-depression, providing scientific guidance for the subsequent optimization of parent combinations.

## 4. Discussion

In this study, semi-diallel hybridization was performed on representative cold-region materials and materials with unverified adaptability (a total of 10 parents), aiming to screen excellent hybrid combinations suitable for cold-region cultivation, obtain information on target agronomic traits, and provide a basis for formulating breeding strategies for trait improvement. The results of variance analysis showed that there were significant differences among different genotypes (*p* < 0.05), indicating that the materials had significant genetic variation and had the potential for further improvement. The main environmental effects (ENV) of all traits except ear length and stem thickness were highly significant (*p* < 0.01), indicating that environmental factors played a dominant role in their expression. This finding is consistent with the pattern observed in multi-environment experiments, that is, environmental variation usually accounts for the largest share of the total variation of traits [20]. At the same time, the genotype × environment interaction effects (G × E) of traits such as grain yield, number of rows per ear, and water content did not reach a significant level (*p* > 0.05). Previous studies have pointed out that when different genotypes have similar response patterns to environmental changes (i.e., their relative performance rankings remain essentially unchanged in different environments), the contribution of G × E interactions to the total variation is often not significant [21]. In other words, the genotypes in this study had similar response patterns to environmental changes, and the traits showed high stability and broad adaptability, which is conducive to application under different environmental conditions. In addition, in the same environment, the differences between different genotypes in repeated experiments (Rep) were not significant, which indirectly confirmed that the main effect of genotype was relatively weak (i.e., the contribution of genotype to phenotypic variation was small), further emphasizing the dominant role of environmental factors in driving trait variation.

The combining ability analysis revealed that, in the dataset combined across four environments, the GCA and SCA effects for all traits were highly significant, indicating that these traits are jointly governed by additive and non-additive genetic effects. Fan [22] and Woldu [23] also reached similar conclusions in their maize studies. For all traits except grain yield and kernel rows per ear, the GCA mean squares exceeded the corresponding SCA mean squares, indicating that additive effects predominantly govern these traits. Although additive variance drives most of the variation, dominance and overdominance also play significant roles in the genetic architecture. The predominance of additive effects suggests that selection-based breeding will be particularly effective for trait improvement. Kamara’s findings indicate that kernel row number is primarily driven by non-additive effects, whereas plant height and ear height are governed by additive effects, in agreement with our study—though their results for grain yield differ [24]. Notably, other work has shown that under drought conditions, yield is mainly controlled by additive gene action, while in well-watered environments, non-additive effects predominate [25]. In their study on the interaction between GCA × E and SCA × E in maize, Nass [26] and Auguira [27] found that GCA × E showed significant effects on all traits, while the interaction effect of SCA × E did not reach the extremely significant level. This result is highly consistent with the findings of this study. Traits with Baker’s ratios above 0.80—namely moisture content, ear height, and husk leaf number—are largely under additive control, meaning additive genetic variance contributes most to their total genetic variation. Moreover, because additive variance correlates closely with heritability, selection-based breeding offers an effective route to improve these traits. In this study, nearly all hybrid combinations showed significant SCA effects (*p* < 0.05 or *p* < 0.01) for yield, grain moisture content, plant height, ear height, ear length, husk leaf number, stalk diameter, stem node number, and kernel rows, indicating active non-additive genetic effects. This pattern is common in maize hybrid research. Iqbal [28] also reported highly significant SCA for almost all traits in a Griffing full-diallel study, underscoring the importance of dominance genes. Likewise, Hasan [29] confirmed that SCA for yield and its component traits remains significant, supporting the validity of our results. Studies have shown a strong genetic correlation between a parent’s general combining ability (GCA) and the specific combining ability (SCA) of its hybrids. GCA is primarily governed by additive genetic variance, and because additive effects have high heritability, they can be fixed through selection. Consequently, traits predominantly controlled by additive gene action are more readily improved by selection in breeding programs [30]. Accordingly, differences in GCA effects among parental lines have a major influence on parental pairing decisions in breeding. In combining ability analysis, any parent exhibiting a highly significant positive GCA effect is regarded as a superior general combiner, positively influencing the trait performance of its hybrid progeny [31]. In this study, GCA effects for each trait were widely distributed among genotypes, indicating that no single parent held an absolute GCA advantage across all target traits. Parent P9 exhibited the highest GCA effects for grain yield and grain moisture content, suggesting it carries favorable additive alleles for these traits. Parent P6 showed the strongest GCA effects for ear length and husk leaf number, making positive contributions to hybrid performance for those traits.

In studies of the genetic control of maize yield components, combining parents with different levels of combining ability offers diversified strategies for improving yield and stability. Iqbal’s [32] study showed that, compared with other combinations, hybrids produced by crossing high-GCA parents with low-GCA parents exhibited superior performance in grain yield and its component traits. Jinks [33] pointed out that in crosses between high- and low-GCA parents, the interaction of overdominance and heterosis effects often generates significant specific combining ability (SCA); in further analyses of F_2_ and backcross generations, he also explained that dominance genes can cancel out with their modifiers, sometimes leading to unfavorable SCA effects. Interestingly, when two low-GCA parents are combined, gene complementation can offset the weaknesses of each parent, yielding unexpectedly high SCA effects [34]. In summary, by balancing additive and non-additive gene effects in both high-GCA × low-GCA and low-GCA × low-GCA crosses, theoretical support and practical guidance can be provided for designing maize hybrid combinations.

The present study identified several parental combinations, including P1 × P9, P2 × P5, P3 × P10, P4 × P6, P5 × P8, P6 × P9, P7 × P10, and P8 × P10, that exhibited significant specific combining ability (SCA) for critical agronomic traits such as yield, ear length, stem diameter, and kernel number per row under cold conditions. These combinations have high potential value for breeding applications. Previous studies have indicated that heterosis (hybrid vigor) arises from dominance, overdominance, and epistatic gene interactions, and the intensity of these effects is closely associated with genetic diversity between parental lines. Further genomic-level analyses revealed that maize yield and its component traits are influenced by additive effects, but predominantly controlled by non-additive genetic effects [35]. The results from this study further confirm that non-additive genetic effects dominate in controlling most target traits. Hence, in practical breeding activities, parental combinations exhibiting significant specific combining ability (SCA) should be preferentially selected to fully exploit heterosis, facilitating the development of high-yielding and high-quality maize varieties.

Studies using the Hayman method have shown that all quantitative traits of maize are regulated by both additive and non-additive genes. According to Hayman’s analysis, the comparison of H_1_, H_2_, and additive variance D of different traits reveals the uneven distribution of genotype structure. In most traits, H_2_ < H_1_ and H_2_/4H_1_ < 0.25, indicating that dominant alleles tend to accumulate. These findings are consistent with the results reported by Ali and Hussain [36]. For most traits, both H_1_ and H_2_ were negative, and |H_1_| > |D|, indicating that non-additive effects predominate in the genetic variance. This conclusion is consistent with Geetha and Jayaraman’s [37] analysis of maize line hybrids, where dominance effects (H_1_, H_2_) typically exceed additive effects for traits such as plant height and kernels per row. In addition, the average dominance of all traits (H_1_/D)^1/2 is greater than 1, which means that the genetic effect tends to be overdominant or dominant; this is consistent with the “overdominant” genetic tendency of maize traits reported in many studies. In contrast, no trait showed (H_1_/D)^1/2 = 0, that is, there are no traits with pure additive regulation. The genetic variance components D and H_1_ are mostly negative and |H_1_| > D, which again supports the dominance of non-additive gene effects. In terms of genetic parameters, the h^2^ of most traits in this study is at a medium or low level, which indicates that the environmental effect is strong and the selection progress may be slow. It is worth noting that plant height, ear position, and number of grains per ear row showed abnormally high h^2^ estimates (16,295.68%, 4037.08%, and 130.16%, respectively), which may be attributed to the negative value of additive variance D or the small error estimate leading to exaggerated genetic variance, a phenomenon also reported in previous studies on complex quantitative traits [16]. Therefore, maize breeding should focus on making full use of heterosis and improving trait performance through reasonable gene combination and hybrid combination improvement strategies.

Regression analysis indicated that the inheritance of grain moisture content follows an additive–dominance model: its regression coefficient is statistically significant from 0 but not significantly different from 1, implying no detectable non-allelic interactions. This finding aligns with the work of Ali [36] on maize grain moisture content, who likewise reported a regression coefficient significantly different from 0 yet close to 1, with no evidence of significant non-allelic interactions. On the contrary, the regression coefficients of traits such as bract number, ear length, number of grains per row, and plant height in the F_1_ generation all deviated significantly from 0 and 1, indicating that the inheritance of these traits was affected by significant allelic interactions (such as dominance or epistasis). Srdić [38] found that the trait of kernel number per row in maize was mainly epistatic (overdominant) in the genetic analysis; Rafiq [39] also reported that plant height was controlled by overdominance and complementary gene interaction; for other traits, the regression coefficient deviated significantly from 1 but not significantly from 0, suggesting that its inheritance was mainly dominated by dominant effects or non-allelic gene interactions. The study by Srdić [38] also showed that the dominant effect was dominant in many maize yield-related traits. Overall, these findings are consistent with the existing literature, and the inheritance patterns of each trait clearly show the different degrees of effects of alleles or non-allelic interactions.

The Vr-Wr regression results of this study are consistent with previous maize Vr-Wr studies: Padma Lay [40] observed that the intercept of yield and related grain traits in multi-point hybrid materials was significantly lower than zero, reflecting an overdominant effect; Zare [41] pointed out that the yield and plant height are affected by overdominance in his analysis of complete diallel hybrids in maize; Yi’s [42] QTL study also showed that grain traits such as row kernels and ear weight were rich in overdominant loci; other studies have shown that the main effect gene controlling the number of leaves above the ear position is mainly dominant [43]. In summary, the overdominant gene action of yield-related traits provides a genetic basis for hybrid vigor, while the additive or partially dominant characteristics of stem node number and plant type traits suggest that the use of both dominant and additive effects can be taken into account in breeding selection to optimize the performance of hybrid combinations.

## 5. Conclusions

In this study, yield-related traits of cold-region maize and potential cold-region-adapted maize were analyzed. The results showed that these traits were genetically controlled by additive and non-additive effects. Combining ability analysis based on four cold-region environments showed that all traits showed significant general combining ability (GCA) and specific combining ability (SCA). The root mean square of GCA of all traits except yield and number of kernels per row was greater than the root mean square of SCA, indicating that these traits were mainly determined by additive genetic effects. Both parents, P6 and P9, have a good genetic basis for improving the yield traits of cold-region maize and are suitable as excellent parents. Further analysis showed that hybrid combinations such as P1 × P9, P2 × P5, P3 × P10, P4 × P6, P5 × P8, P6 × P9, P7 × P10, and P8 × P10 showed good heterosis and strong breeding potential, and were suitable for subsequent hybrid breeding of cold-region maize. In addition, yield, ear length, number of grains per row, water content, and plant height showed obvious dominant effects, while stem diameter, number of stem nodes, and ear position showed incomplete dominant effects. Parent P1 had more recessive genes in yield traits and more dominant genes in number of grains per row, number of stem nodes, and ear position; P3 had more dominant genes in yield and water content and more recessive genes in number of stem nodes and ear position; P7 had more recessive genes in most traits; and P9 had more dominant genes in most traits. P9 and P6 represent excellent parental germplasm, among which are hybrid combinations. Becker ratio analysis showed that the selection method based on additive effects can significantly improve the genetic improvement efficiency of yield traits of cold-region maize.

## Figures and Tables

**Table 1:** Experimental materials.

Parent	Genotype	Cold-Region Type	Parent	Genotype	Cold-Region Type
P1	K10	Cold-region maize	P6	DE1 (male parent)	Cold-region maize
P2	KF298	Cold-region maize	P7	DE3 (female parent)	Cold-region maize
P3	Dong46	Cold-region maize	P8	DE3 (male parent)	Cold-region maize
P4	Jing7	Cold-adapted (potential)	P9	FA5-1	Cold-region maize
P5	He344	Cold-adapted (potential)	P10	FA5-5	Cold-region maize

**Table 2:** Combined analysis of variance for nine traits of ten parents and their hybrid combinations across four environments.

	Env	Genotype	Rep	G×E	Error	CV
DF	3	54	2	54	440	
EL	2.65	24.86 **	7.1	3.33	3.19	44.81
SD	20.32	17.17 **	11.33	1.82	6.31	38.17
KR	270.86 **	114.38 **	38.11	18.72	15.59	20.26
NN	165.67 **	10.01 **	17.04 **	1.72	2.03	58.4
NB	119.05 **	9.69 **	2.63	5.55 **	1.25	63.55
YLD	123.86 **	32.68 **	0.08	4.8	6.48	33.98
MC	71.15 **	13.71 **	13.79	6.19	5.46	39.81
EH	7041.87 **	3786.13 **	10.32	216.10 **	124.95	4.75
PH	1711.87 **	12,736.10 **	323.02	605.43 **	197.39	2.82

EL: ear length; SD: stem diameter; KR: kernel rows per ear; NN: number of nodes; NB: number of bracts; YLD: yield; MC: moisture content; EH: ear height; PH: plant height. CV: coefficient of variation.; ** *p* < 0.01.

**Table 3:** Mean performance of parental lines across four environments.

	P1	P2	P3	P4	P5	P6	P7	P8	P9	P10
EL	63.68	50.98	78.75	60.07	66.95	52.72	74.73	69.92	82.7	64.98
SD	21.34	22.48	19.94	20.52	21.74	21.03	21.33	22.66	20.22	22.35
KR	25.56	26.71	29.11	25.83	29.87	27.42	29.44	27.09	29.49	26.88
NN	10.4	10.63	11.92	11.1	11.33	10.53	11.07	11.53	11.75	11.08
NB	9.42	9.1	9.7	8.63	9.35	9.9	8.57	9	9.08	9
YLD	7.26	5.5	5.64	4.75	5.79	5.55	5.52	5.45	6.05	4.52
MC	24.38	23.76	22.26	23.65	21.27	22.67	24.81	24.08	25.56	24.06
EH	63.68	50.98	78.75	60.07	66.95	52.72	74.73	69.92	82.7	64.98
PH	214	142.2	179.42	171.33	198.62	181.75	221.2	227.8	212.83	194.8

EL: ear length; SD: stem diameter; KR: kernel rows per ear; NN: number of nodes; NB: number of bracts; YLD: yield; MC: moisture content; EH: ear height; PH: plant height.

**Table 4:** Analysis of variance for general combining ability and specific combining ability.

	GCA	SCA	GCA × E	SCA × E	Baker Ratio	GCA/SAC
DF	9	44	9	44		
YLD	1.89 **	2.89 **	41.22 **	1.57	0.57	0.65
MC	2.70 **	0.83 **	23.71 *	2.06	0.87	3.25
PH	1351 **	1003 **	270.62	201.80	0.73	1.35
EH	547 **	269 **	2747 **	72.03	0.8	2.03
EL	2.13 **	2.05 **	0.88	1.11	0.68	1.04
NB	2.86 **	0.39 **	36.68 **	1.84 **	0.94	7.33
SD	1.90 **	1.33 **	6.77	0.60	0.74	1.43
NN	1.38 **	0.72 **	55.22 **	0.57	0.79	1.92
KR	8.66 **	9.70 **	90.28 **	6.23	0.64	0.89

EL: ear length; SD: stem diameter; KR: kernel rows per ear; NN: number of nodes; NB: number of bracts; YLD: yield; MC: moisture content; EH: ear height; PH: plant height. * *p* < 0.05; ** *p* < 0.01.

**Table 5:** General combining ability (GCA) effect values.

	YLD	MC	PH	EH	EL	NB	SD	NN	KR
P1	−0.08	0.22	14.60 **	3.26 **	0.36 *	0.21	0.03	0.14	0.5
P2	−0.05	0.38	−19.18 **	−7.66 **	0.26	−0.09	0.56 **	−0.22	−0.25
P3	−0.13	−0.16	−1.91	9.91 **	0.09	0.08	−0.71 **	0.24	0.77 *
P4	−0.02	0.14	−14.02 **	−4.19 **	−0.83 **	−0.18	−0.01	−0.04	−1.36 **
P5	−0.08	−0.83 **	1.86	0.23	0.43 **	0.11	0.34	0.03	1.37 **
P6	−0.26	−0.58 *	−7.28 **	−13.03 **	0.44 **	1.21 **	0.47 *	−0.86 **	−0.06
P7	−0.14	0.01	8.40 **	3.90 **	0.11	−0.61 **	−0.34	0.06	0.07
P8	0.49	0.37	6.99 **	−1.22	−0.09	−0.1	0	0.36 *	0.56
P9	0.86 **	0.73 **	4.65 **	4.66 **	−0.32	−0.27 *	−0.40 *	0.16	−0.44
P10	−0.57 *	−0.28	5.91 **	4.14 **	0.46 **	0.36 **	0.05	0.1	1.16 **

EL: ear length; SD: stem diameter; KR: kernel rows per ear; NN: number of nodes; NB: number of bracts; YLD: yield; MC: moisture content; EH: ear height; PH: plant height. * *p* < 0.05; ** *p* < 0.01.

**Table 6:** Specific combining ability (SCA) effect values.

	YLD	MC	PH	EH	EL	NB	SD	NN	KR
P1 × P2	−1.30 **	−0.89 **	22.82 **	10.04 **	0.84 **	0.23 **	1.36 **	0.41 **	1.77 **
P1 × P3	1.28 **	0.55 **	1.75 **	2.31 **	0.63 **	−0.08	−0.43 **	0.05	2.34 **
P1 × P4	0.11	−0.06	16.11 **	9.79 **	0.75 **	−0.13 **	0.11	0.82 **	0.18
P1 × P5	−1.25 **	0.69 **	21.88 **	8.12 **	1.35 **	0.01	1.12 **	0.26 **	3.11 **
P1 × P6	0.27 **	0.89 **	10.83 **	2.11 **	0.43 **	0.48 **	0.45 **	0.15 **	0.68 **
P1 × P7	2.21 **	−0.22 **	12.80 **	8.32 **	0.73 **	−0.16 **	−0.77 **	0.48 **	2.86 **
P1 × P8	−0.70 **	0.83 **	8.69 **	2.76 **	0.23 **	1.56 **	0.73 **	−0.10 *	1.70 **
P1 × P9	−0.09	0.59 **	18.76 **	16.23 **	1.24 **	0.16 **	1.47 **	0.31 **	2.23 **
P1 × P10	0.62 **	−0.75 **	21.83 **	17.10 **	0.29 **	−0.53 **	−0.89 **	2.18 **	2.08 **
P2 × P3	1.14 **	1.20 **	16.63 **	4.30 **	−0.17 **	0.34 **	0.01	0.43 **	0.09
P2 × P4	0.30 **	0.40 **	8.96 **	−1.66 **	0.88 **	−0.04	1.09 **	−0.16 **	1.01 **
P2 × P5	0.42 **	0.53 **	25.11 **	9.44 **	0.97 **	0.21 **	−0.40 **	0.45 **	2.03 **
P2 × P6	0.50 **	0.21 **	5.00 **	6.44 **	0.60 **	0.63 **	−0.48 **	0.09 *	0.06
P2 × P7	0.14	−0.17 *	16.67 **	5.09 **	−0.02	0.52 **	0.28 **	0.64 **	0.58 **
P2 × P8	0.40 **	1.00 **	9.64 **	4.36 **	0.91 **	0.07	−0.44 **	0.46 **	2.86 **
P2 × P9	1.93 **	0.38 **	7.86 **	1.48 **	1.02 **	−0.49 **	1.98 **	−0.18 **	1.18 **
P2 × P10	1.30 **	0.87 **	31.26 **	18.99 **	1.14 **	−0.47 **	−0.43 **	0.52 **	2.04 **
P3 × P4	0.83 **	0.99 **	21.69 **	17.71 **	0.55 **	−0.26 **	0.13 *	0.22 **	1.03 **
P3 × P5	1.10 **	0.31 **	19.96 **	6.13 **	0.39 **	0.10 **	0	0.56 **	−0.73 **
P3 × P6	−1.37 **	0.35 **	20.01 **	3.76 **	0.12 *	0.29 **	1.14 **	0.15 **	0.15
P3 × P7	−1.92 **	−0.17 *	18.08 **	16.44 **	1.22 **	0.05	0.92 **	0.32 **	1.69 **
P3 × P8	−0.03	0.91 **	1.85 **	0.46	0.80 **	−0.37 **	−0.60 **	−0.12 *	3.34 **
P3 × P9	2.39 **	0.12	16.02 **	9.53 **	0.13 **	0	1.48 **	0.05	0.79 **
P3 × P10	0.77 **	0.11	22.63 **	12.57 **	1.17 **	0.39 **	0.34 **	0.28 **	2.23 **
P4 × P5	1.69 **	0.81 **	8.55 **	6.97 **	1.02 **	0.58 **	1.01 **	0.36 **	0.47 **
P4 × P6	1.03 **	0.68 **	17.19 **	8.07 **	1.29 **	0.84 **	0.93 **	0.01	2.65 **
P4 × P7	−0.60 **	−0.26 **	1.06 *	−0.21	0.53 **	0.13 **	−0.36 **	0.45 **	0.55 **
P4 × P8	2.61 **	0.45 **	6.15 **	9.57 **	−0.07	0.61 **	0.74 **	0.43 **	0.81 **
P4 × P9	0.30 **	−0.08	10.93 **	−1.32 **	0.86 **	−0.53 **	0.69 **	−0.17 **	1.80 **
P4 × P10	0.16	−0.14 *	15.70 **	5.30 **	0	0.35 **	0.28 **	0.48 **	0.45 **
P5 × P6	−1.83 **	0.94 **	7.23 **	1.92 **	0.57 **	−0.42 **	0.58 **	−0.41 **	1.52 **
P5 × P7	1.39 **	0.67 **	4.21 **	5.97 **	0.15 **	0.17 **	−0.04	0.31 **	0.90 **
P5 × P8	1.49 **	0.22 **	−0.17	−0.34	0.35 **	−0.10 *	0.86 **	0.21 **	1.35 **
P5 × P9	0.85 **	−0.25 **	15.10 **	11.19 **	0.89 **	0.44 **	−0.32 **	0.44 **	0.37 **
P5 × P10	0.22 **	−0.25 **	13.42 **	8.72 **	0.91 **	0.28 **	0.74 **	0.07	2.78 **
P6 × P7	0.49 **	1.06 **	13.38 **	5.87 **	0.36 **	0.39 **	0.32 **	0.70 **	2.34 **
P6 × P8	1.14 **	−0.42 **	6.34 **	2.54 **	1.21 **	0.51 **	1.02 **	−0.02	1.29 **
P6 × P9	1.34 **	−0.70 **	18.13 **	0.78 *	0.66 **	1.30 **	0.80 **	−0.20 **	2.29 **
P6 × P10	2.30 **	−1.15 **	14.36 **	2.03 **	0.32 **	0.54 **	0.77 **	−0.16 **	0
P7 × P8	1.26 **	−0.84 **	12.27 **	5.81 **	0.07	−0.50 **	−0.68 **	0.05	0.86 **
P7 × P9	1.27 **	−0.83 **	7.04 **	3.51 **	1.02 **	−0.55 **	0.36 **	0.10 *	−0.61 **
P7 × P10	0.16	0.72 **	10.78 **	6.42 **	−0.42 **	−0.09 *	1.61 **	−0.14 **	−1.70 **
P8 × P9	0.55 **	0.95 **	13.88 **	8.81 **	−0.33 **	−0.16 **	−1.42 **	1.93 **	−0.32 **
P8 × P10	0.33 **	−0.27 **	18.82 **	12.42 **	0.64 **	−0.47 **	0.18 **	0.31 **	2.21 **
P9 × P10	−1.20 **	1.16 **	−9.68 **	−5.88 **	−1.43 **	0.10 *	−1.39 **	−0.34 **	−2.42 **

EL: ear length; SD: stem diameter; KR: kernel rows per ear; NN: number of nodes; NB: number of bracts; YLD: yield; MC: moisture content; EH: ear height; PH: plant height * *p* < 0.05; ** *p* < 0.01.

**Table 7:** Hayman genetic parameter analysis.

	YLD	NB	EL	SD	KR	NN	MC	EH	PH
(H_1_/D)^1/2	1.17	1.35	1	1.36	1	1.3	1.79	5.46	1.97
H_2_/4H_1_	0.12	0.18	0.06	0.15	0.09	0.15	0.17	0.25	0.24
h^2^/H_2_	−6.37	−0.59	−48	−3.39	−28.84	−4.53	−0.64	8.83	8.58
$h_{N}^{2}$	0	0.29	0.04	0	0.03	0.07	0	0.31	0.22
r	−0.53	−0.52	−0.96	−0.49	−0.85	−0.3	−0.53	−0.64	−0.96
D	−5.64 **	1.37 **	−2.15 **	2.47 **	−11.57 **	1.50 **	2.98 **	15.27	489.79
H_1_	−7.80 **	2.53 **	−2.16 **	4.62 **	−11.64 *	2.55 **	9.62 **	455.96	1917.07
H_2_	−4.02 **	1.91 **	−0.59 **	2.77 **	−4.51	1.57 **	6.77 **	457.18	1897.53
h^2^	25.63 **	1.13 **	28.78 **	9.43 **	130.16 **	7.16 **	4.35 **	4037.08	16,295.68
E	6.19 **	1.56 **	2.80 **	3.38 **	14.14 **	1.77 **	4.59 **	93	201

EL: ear length; SD: stem diameter; KR: kernel rows per ear; NN: number of nodes; NB: number of bracts; YLD: yield; MC: moisture content; EH: ear height; PH: plant height (H1/D)1/2: average dominance degree; H2/4H1: ratio of the positive and negative gene effects in all parents; h2/H2: the number of dominant gene loci; D: additive effect; $h_{N}^{2}$ : narrow-sense heritability; r: direction of dominance; H2: dominance variance component; H1: the variance due to the dominance effect of genes; h2: dominance effect; E: environmental variance; * *p* < 0.05; ** *p* < 0.01.

**Table 8:** Hypothesis testing of the Hayman model.

	YLD	NB	EL	SD	KR	NN	MC	EH	PH
t^2^	2.082	11.88 **	3.46	2.27	1.32	25.20 **	0.19	3.53	4.01 *
B = 0	0.724	2.72 *	3.66 **	0.55	3.36 **	1.18	4.92 **	1.11	5.86 **
B = 1	3.975 **	5.94 **	3.18 **	4.2 **	2.32 *	9.71 **	1.21	4.34 **	2.92 **
b	0.154	0.31	0.53	0.11	0.59	0.1	0.8	0.2	0.66

EL: ear length; SD: stem diameter; KR: kernel rows per ear; NN: number of nodes; NB: number of bracts; YLD: yield; MC: moisture content; EH: ear height; PH: plant height * *p* < 0.05; ** *p* < 0.01. t^2^: test of validity of hypothesis.

## Data Availability

The data that support the findings of this study are available from the corresponding author upon reasonable request.

## Abbreviations

The following abbreviations are used in this manuscript:

GCA	General combining ability
SCA	Specific combining ability
